# Hypnotism in the Treatment of Dyspareunia

**Published:** 1909-10-02

**Authors:** Douglas Bryan

**Affiliations:** Leicester


### HYPNOTISM IN THE TREATMENT OF DYSPAREUNIA.

Sir,—In your issue of The Hospital of September 25, 1909, in "Notes on Dyspareunia," I notice at the end of the article the following passage: "Where no local lesion exists and the vaginal entrance is wide, dyspareunia seems practically incurable." This assertion is evidently made by one who does not know of treatment by hypnotism and suggestion, for its efficacy in this very troublesome condition is now well established by a number of very successful recorded cases. I will give the notes of one occurring in my own practice.

In April 1907 I delivered a patient of her first child; forceps were used, and there was a slight perineal tear, necessitating one suture. The tear healed by the tenth day. A month after the delivery coitus was attempted, but abandoned on account of the great pain caused, and subsequent attempts were equally unsuccessful. The patient ever since her marriage had had a good deal of pain, but not nearly as severe as she now experienced. About three months after the birth of her child she consulted me in order to obtain some relief. On examination I found she was most tender just inside the vagina on the right side, and there appeared to be two or three small patches on the mucous membrane which were very painful. I tried a number of local remedies without any satisfactory result, and then advised her to try hypnotism and suggestion; but she would not hear of it. I next suggested a consultation, and the surgeon called in advised cauterising all patches on the mucous membrane. This was done under a general anæsthetic, but no good resulted. The patient left off all treatment for a time, but the condition remained the same. At last, fearing that her domestic happiness was becoming endangered—as is so likely to be the case with this condition—she consulted me in the latter part of 1908; and I again advised her to try hypnotism and suggestion, after pointing out to her the harmlessness of the treatment and its possibilities. She consented to give it a trial, and a few days later came with her husband for a sitting. I early succeeded in inducing a light degree of hypnosis and made the appropriate suggestions. In all the treatment lasted a quarter of an hour. Next day she came joyfully to tell me that the treatment had been quite successful; she had had no pain during the act. She required no further treatment, and has remained quite well up to the present.

Now whether this dyspareunia is put down to local lesion or not—and I do not consider any local lesion was causing it—the fact remains that by hypnotism and suggestion the patient was cured, and I will further add she was not a hysterical subject. I could quote further cases to prove my contention that there is a treatment for these cases where no local lesion exists. Yours, etc.,

Douglas Bryan, M.R.C.S., L.R.C.P.

Leicester, September 25.

